# "Is Cybermedicine Killing You?" — University College London (UCL) Media Strategy Explained: Author's Reply

**DOI:** 10.2196/jmir.7.4.e44

**Published:** 2005-07-28

**Authors:** Gunther Eysenbach

## Author's Response

The UCL media strategy as described in the letter of Fourniol has been understood by us and has been accurately described in our editorial [1]. In fact, it is exactly this strategy which has been criticized as insufficient (some may say even unethical). It would have been more in the public's interest to immediately and unambiguously disseminate the fact that these major errors and misinformation occurred (and their magnitude), rather than waiting many months for the revision to be published. The strategy of the UCL media office is akin to a car manufacturer not recalling a faulty vehicle immediately after errors become apparent, but waiting first for a new model to be developed before starting a campaign to exchange the flawed model.

## References

[1] Eysenbach Gunther, Kummervold Per Egil (2005). "Is cybermedicine killing you?" - the story of a Cochrane disaster. J Med Internet Res.

